# Laboratory Evaluation of the Use of Florida Washed Shell in Open-Graded Asphalt Mixtures

**DOI:** 10.3390/ma14227060

**Published:** 2021-11-21

**Authors:** Mohammad Alharthai, Qing Lu, Ahmed Elnihum, Asad Elmagarhe

**Affiliations:** 1 Department of Civil and Environmental Engineering, University of South Florida, Tampa, FL 33620, USA; elnihum@usf.edu (A.E.); elmagarhe@usf.edu (A.E.); 2 Department of Civil Engineering, Najran University, Najran 66446, Saudi Arabia

**Keywords:** open-graded asphalt mixture, aggregate gradation, Florida washed shell, sustainable pavement materials, Marshall stability, indirect tensile strength, Cantabro test

## Abstract

This study investigates the substitution of conventional aggregate with a Florida washed shell in open-graded asphalt mixtures and evaluates the optimal substitution percentage in aggregate gradations of various nominal maximum aggregate sizes (NMASs) (i.e., 4.75, 9.5, and 12.5 mm). Laboratory experiments were performed on open-graded asphalt mixture specimens with the coarse aggregate of sizes between 2.36 and 12.5 mm being replaced by the Florida washed shell at various percentages (0, 15, 30, 45, and 100%). Specimen properties relevant to the performance of open-graded asphalt mixtures in the field were tested, evaluated, and compared. Specifically, a Marshall stability test, Cantabro test, indirect tensile strength test, air void content test, and permeability test were conducted to evaluate the strength, resistance to raveling, cracking resistance, void content, and permeability of open-graded asphalt mixtures. The results show that there is no significant difference in the Marshall stability and indirect tensile strength when the coarse aggregates are replaced with Florida washed shell. This study also found that the optimum percentages of Florida washed shell in open-graded asphalt mixture were 15, 30, and 45% for 12.5, 9.5, and 4.75 mm NMAS gradations, respectively.

## 1. Introduction

Open-graded asphalt mixture has been used by many road agencies in the United States since 1950 [1]. It features a large, interconnected air void system in the compacted mixture, which is achieved through the use of an open aggregate gradation. When the open-graded asphalt mixture is used in a porous pavement structure, it allows water to infiltrate into the base and subgrade layers to reduce storm water runoff and remove some contaminants from the runoff. When the open-graded asphalt mixture is used as the surface course of a conventional flexible pavement, it facilitates quick drainage of surface water to roadsides to reduce hydroplaning potentials of vehicles traveling on wet pavements.

Aggregate (as the structural skeleton of the mixture) and asphalt binder (as glue for the aggregate) are the two major components of an open-graded or porous asphalt mixture. When the mixture is placed and compacted at a high temperature, it is referred to as a hot mix asphalt (HMA). Generally, the aggregate constitutes about 90% of the HMA volume. Therefore, the properties of the aggregate play a major role in the performance of asphalt pavements [2]. Based on previous studies [3,4,5,6], the gradation and nominal maximum aggregate size (NMAS) of aggregates have a significant effect on the durability and permeability of asphalt pavement surface. Aggregate gradation is the distribution of aggregate sizes expressed as mass percentage passing through a series of sieves with various sizes. NMAS is the largest sieve size that retains some of the aggregate particles but generally less than 10 percent by weight [7].

Natural aggregate is the largest source of materials in building and pavement construction. For example, two billion and 400 million tons of aggregates are consumed each year in the United States and France, respectively [8,9]. In recent decades, due to the depletion of many good aggregate sources and increased negative environmental impact from the excavation and processing of natural aggregates, many studies have considered the replacement of virgin aggregates in asphalt and cement concrete pavements by recycled aggregates, slag, reclaimed asphalt pavement (RAP) materials, seashell, and many other alternatives [8,10,11,12,13,14] Cost reduction is another major benefit from replacement of natural aggregates in asphalt mixtures [11]. For example, in regions where the source of natural aggregate is limited, aggregates have to be transported from a long distance, which may significantly increase the aggregate cost [14]. This cost may be reduced by replacing the aggregates with locally available materials. For coastal regions, such as Florida, one commonly available material is seashell. The seashells abundant on beaches come from sea animals with shells that died naturally. The consumption of shellfish by humans worldwide also generates thousands of tons of seashells. For example, oyster waste is considered a problem in Asian countries such as China, Taiwan, and South Korea, where 370–700 kg of waste is produced from every 1000 kg of oyster shell [15]. Landfill is the most common method of managing these seashells, but it has negative environmental impact [10,14]. An untreated seashell landfill can produce an unpleasant odor due to the dissolution of the remaining tissue in the shells or due the conversion of salts into gases such as H_2_S, NH_3_, and amines by microbes [16].

Most previous studies have focused on reusing washed shell in certain areas, such as biochemical technology, water-quality refining, and soil enhancement [17]. Recently, however, a limited number of studies have investigated the use of seashell material in asphalt mixtures as coarse aggregate or filler [12,18]. It was found that, when seashell material was used as a filler in HMA, it increased the stability and stripping resistance of the asphalt mixture [18]. There is, however, little research on more detailed properties and the design of porous asphalt mixtures containing seashells as aggregate. Due to the high angularity of broken seashell, it is assumed that the inclusion of seashell material in open-graded asphalt mixtures would contribute to an aggregate-interlocking skeleton structure for good mixture stability and desirable interconnected air void system. Currently, there is no guidance on the use, particularly at a high percentage, of seashell as coarse aggregates in open-graded asphalt mixtures.

This study intends to investigate and evaluate the replacement of coarse aggregate in open-graded asphalt mixtures with seashell. Specifically, a seashell product that is mined from Florida beaches, the Florida washed shell, is included in the study. The influence of aggregate gradations of various NMASs (i.e., 4.75, 9.5, and 12.5 mm) on the optimum percent of Florida washed shell in open-graded asphalt mixtures is also investigated.

To achieve the aims of this study, asphalt mixture specimens are prepared and evaluated with and without Florida washed shell in the laboratory. The materials, mixture preparation, compaction method, and performance tests are described as follows.

## 2. Materials

Three types of materials are mainly used in the experiments: asphalt binder, aggregate, and seashell.

### 2.1. Asphalt Binder

One Superpave performance-graded (PG) asphalt binder, PG 76-22, was selected for this study. It is a styrene-butadiene-styrene (SBS) modified binder and was obtained from a local asphalt supplier in Tampa, Florida. The PG 76-22 asphalt is suitable to be applied in an area that experiences high temperatures like Florida and Saudi Arabia and is widely used in the open-graded friction course mixtures on Florida highways. Following the test procedures of American Association of State Highway and Transportation Officials (AASHTO) T 49 and AASHTO T 316 [19,20], the 25 °C penetration and 135 °C viscosity of the PG 76-22 asphalt were measured, with average values being 30.7 (0.1 mm) and 1587 mPa·s, respectively. The optimum binder content (OBCs) of the PG 76-22 asphalt in open-graded asphalt mixtures was determined following the test method specified in the Florida test method F-5 885 [21], with the intention to allow as much as possible asphalt binder in the mixture without causing excessive binder draindown during construction.

### 2.2. Aggregate

The aggregate used in this study is of granite type and was obtained from a local pavement construction company in Tampa, Florida. Its resistance to degradation was measured using a Los Angeles testing machine based on the laboratory test method specified in AASHTO T 96 [22] and was found to have an average value of 14.9%.

### 2.3. Florida Washed Shell

**Figure 1:**
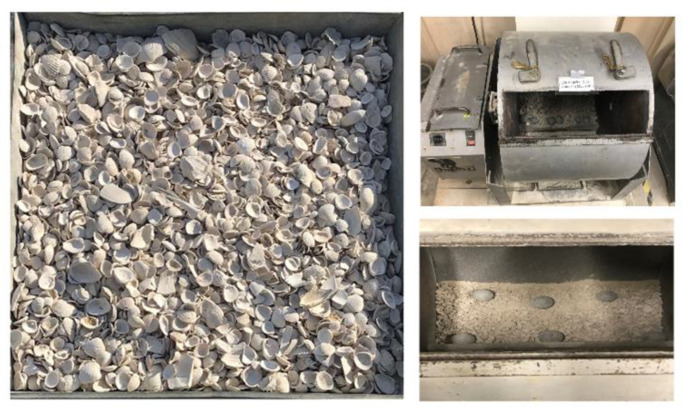
Florida washed shell before and after crushing.

**Figure 2:**
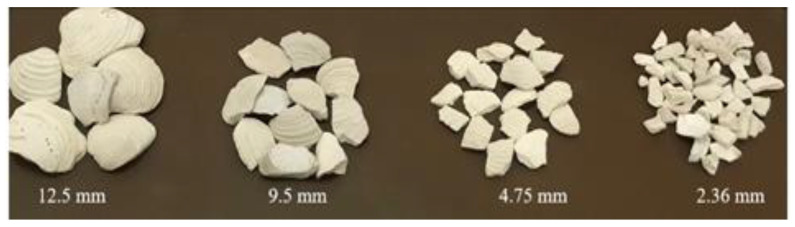
Florida washed shell particles of various sizes used in this study.

The Florida washed shell was provided by a local gravel and washed shell supplier in Tampa, Florida. It had been washed at least twice before it was supplied for use [23]. The shell material mainly consists of calcium carbonate (about 95%), which is similar to the calcium carbonate in limestone, and a small amount of protein. After receiving, the shell was dried under the sun for more than 24 h and then some of them were crushed into small particles in the Los Angeles abrasion machine, as shown in Figure 1. The shell particles were then grouped into four coarse aggregate sizes (12.5, 9.5, 4.75, and 2.36 mm), as shown in Figure 2, for use in the mixture preparation. It can be seen that these seashell particles tend to be flat with irregular shape.

## 3. Mixture Preparation and Compaction Method

This study considered 15 mixture designs, which are the combinations of five contents of Florida washed shell (0, 15, 30, 45, and 100%) and three open-graded aggregate gradations named as NMAS 12.5 mm, NMAS 9.5 mm, and NMAS 4.75 mm, as shown in Table 1. The three gradations have different NMASs (i.e., 4.75, 9.5, and 12.5 mm). The mixtures with 0% shell are, essentially, conventional open-graded asphalt mixtures. The optimum binder contents of the mixtures were determined to be 5.5, 6, and 7.5% (by mass of aggregate) for the 12.5, 9.5, and 4.75 mm NMAS gradations, respectively. The shell particles of sizes in the range of 2.36 mm to 12.5 mm were used to replace the granite aggregates. The percentage of the flat and elongated particles in the combined coarse aggregates (granite and seashell) in various gradations was controlled below 10%.

To prepare test specimens, the proportioned aggregates, asphalt binder, and Florida washed shell were first mixed in a mechanical mixer at 160 ± 2.5 °C for five minutes, then compacted at 155 ± 5.0 °C into cylindrical specimens of a diameter of 101 mm and a nominal height of 63.5 mm using a Marshall compactor. During compaction, 50 blows were applied on each side of the specimens. After compaction, the specimens were allowed to cool down at a room temperature of 25 °C for 24 h and then extracted from the molds.

## 4. Test Methods

### 4.1. Properties of Florida Washed Shell

Since the physical properties of aggregate have a significant impact on the design and performance of asphalt mixtures, it is important to know the properties of washed shell before substituting it for the aggregate. In this study, the test procedures in AASHTO T 85 and AASHTO T 96 [22,24] were followed to measure the bulk specific gravity, saturated surface dry (SSD) bulk specific gravity, apparent specific gravity, and water absorption of Florida washed shell.

### 4.2. Marshall Stability Test

The Marshall stability test was performed according to AASHTO T 245 [25] to measure the stability of asphalt mixture specimens, which is related to the load-carrying capacity of the mixture. Specifically, a compressive load is applied in the diametrical direction of a cylindrical specimen of a diameter of 101 mm (4 inches) at a loading rate of 51 mm/min, and the maximum load is recorded. In this study, this test was conducted at 25 °C instead of 60 °C to prevent excessive creep deformation in the open-graded asphalt mixture specimens during the high temperature conditioning process. A correction factor was applied to the test result when the specimen height differed from the nominal height of test specimens (63.5 mm).

### 4.3. Cantabro Test

The Cantabro test was conducted to measure the raveling resistance of open-graded asphalt mixtures according to American Society for Testing and Materials (ASTM) D 7064 [26]. In the test, one compacted specimen was placed in the Los Angeles abrasion machine drum without abrasion loads (balls), and the drum was rotated at a speed of 30 revolutions per minute for 300 revolutions. The weight of the specimen was measured before and after abrasion in the drum. The test result is the percentage of mass loss, as calculated in Equation (1):

(1) $$L=\frac{\left[{M\;}_{\mathrm{before}\;}-{\;M\;}_{\mathrm{after}}\right]}{\left[{M\;}_{\mathrm{before}}\;\right]}\times 100$$

where L = percentage of mass loss (%); M_before_ = mass of the specimen before being placed into the drum (g); and M_after_ = mass of the specimen after testing (g).

### 4.4. Indirect Tensile Strength Test

The indirect tensile strength test was conducted to evaluate the mixture tensile properties, which are related to cracking resistance, according to ASTM D 6931 [27]. Similar to the Marshall stability test, this test was conducted at 25 °C at a loading rate of 51 mm/min. The specimen dimensions were measured before conducting the test. The indirect tensile strength is calculated according to Equation (2):

(2) $$\mathrm{ITS}=\frac{2000\times P}{\pi \times d\times h}$$

where ITS = indirect tensile strength (kPa); P = applied maximum load (N); d = specimen diameter (mm); and h = specimen height (mm).

### 4.5. Air Void Content

The air void content of each specimen was calculated based on its bulk specific gravity and theoretical maximum specific gravity, as shown in Equation (3). The bulk specific gravity and the theoretical maximum specific gravity were measured in accordance with AASHTO T 275 and AASHTO T 209, respectively [28,29].

(3) $$V_{A}=\left(1-\frac{{G\;}_{\mathrm{mb}}}{{G\;}_{\mathrm{mm}}}\right)\times 100$$

where V_A_ = air void content (%); G_mb_ = bulk specific gravity; and G_mm_ = theoretical maximum specific gravity.

### 4.6. Permeability Test

A falling head permeability test was conducted to determine the water conductivity and the rate of water flow through a compacted asphalt mixture specimen according to FM 5-565 [30]. Specifically, water in a graduated cylinder flows through a specimen and the interval of time taken to reach a known change in the water head is recorded. The coefficient of permeability was calculated using Equation (4):

(4) $$k=\frac{a\;\times \;L}{A\;\times \;t}\times \ln\left(\frac{h_{1}}{h_{2}}\right)\times {\;t}_{c}$$

where k = coefficient of permeability (cm/s); a = the internal cross-sectional area of cylinder (cm^2^); L = specimen thickness (cm); A = the cross-sectional area of the specimen (cm^2^); h_1_ and h_2_ = the initial and final water heads (cm); t = elapsed time for water head change from h1 to h2 (s); and t_c_ = temperature correction coefficient.

## 5. Test Results and Discussion

### 5.1. Florida Washed Shell Properties

The measured aggregate properties are summarized in Table 2. As can be seen, the Florida washed shell has a higher loss value in the Los Angeles abrasion test than the granite aggregate (28.4 versus 14.9%), indicating a lower toughness. Based on the experience gained in the U.S., LA abrasion values of 30 percent or less was recommended for aggregates used in the open-graded friction course [31]. The loss value of the Florida washed shell, therefore, is still within the desired range (less than 30%). Table 2 also shows that the Florida washed shell has lower values of bulk specific gravity and bulk SSD specific gravity, which are 2.600 and 2.669, respectively. The apparent specific gravity of Florida washed shell is slightly higher than that of granite aggregate. The water absorption of Florida washed shell (2.64%) is much higher than that of granite aggregate (0.54%), which indicates that the Florida washed shell will absorb more asphalt binder during mixing [32]. However, Florida washed shell absorption remains within acceptable limits for HMA production in the United States [33].

### 5.2. Marshall Stability Test

**Figure 3:**
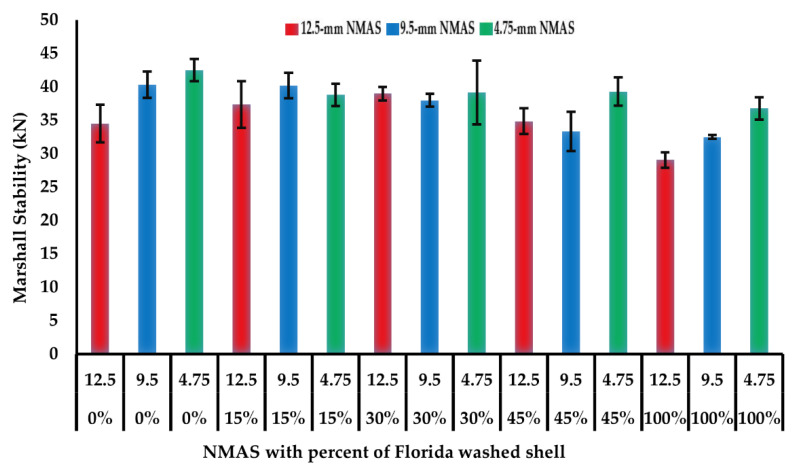
Marshall stability results of open-graded asphalt mixtures at 25 °C.

Marshall stability was conducted to evaluate the strength of open-graded asphalt mixtures with different percentages of Florida washed shell. Figure 3 shows the average and range of one standard deviation of the Marshall stability for 15 mixtures. It can be seen that there is no significant difference in the stability values among mixtures with 0, 15, 30, and 45% of Florida washed shell with 12.5 mm and 4.75 mm NMAS gradations. However, it can be noted that increasing the Florida washed shell percent is negatively affecting the Marshall stability values in mixtures with the 9.5 mm NMAS gradation.

### 5.3. Cantabro Test

**Figure 4:**
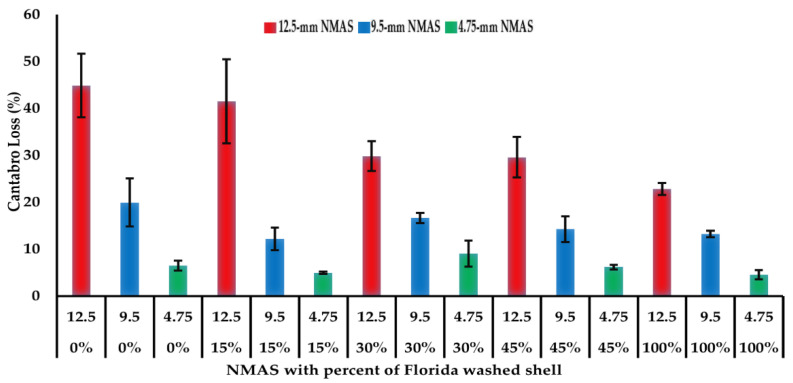
Cantabro loss results of open-graded asphalt mixtures.

The Cantabro test results are summarized in Figure 4 for the 15 open-graded asphalt mixtures. A higher Cantabro loss value indicates a lower resistance to raveling. It can be noticed that, generally, raveling resistance of open-graded asphalt mixtures decreased with the increase of NMAS, and the use of 4.75 mm NMAS significantly increased the raveling resistance, which is consistent with findings from a previous study [4]. For the open-graded asphalt mixtures with the 12.5 mm NMAS gradation, the use of 30, 45, and 100% of Florida washed shell decreased the Cantabro loss compared to 0 and 15% Florida washed shell mixtures. This indicates that replacing a high percent of large-sized aggregates with Florida washed shell can improve the mixture durability (i.e., raveling resistance). For mixtures with a 9.5 mm or 4.75 mm NMAS gradation, the Cantabro loss values were less than 20%, which is the maximum acceptable Cantabro loss value [34].

### 5.4. Indirect Tensile Strength Test

**Figure 5:**
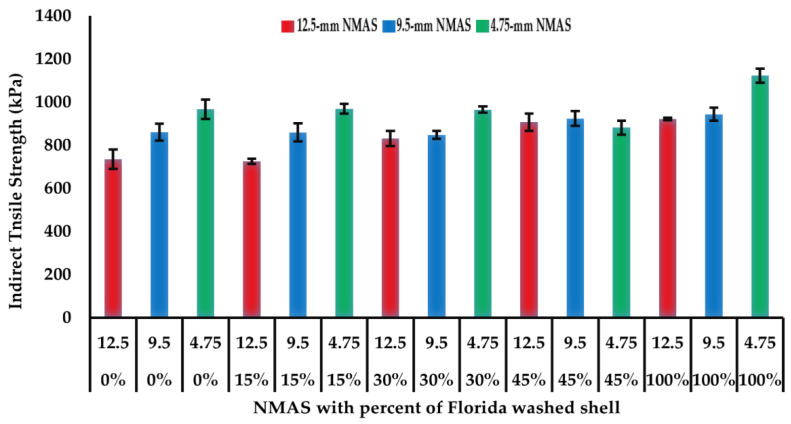
Indirect tensile strength results of open-graded asphalt mixtures at 25 °C.

The indirect tensile strength (ITS) test results for the 15 open-graded asphalt mixtures are shown in Figure 5. There seems to be no significant difference in the indirect tensile strength values when the coarse aggregates are replaced with 15, 30, 45, or 100% Florida washed shell. It can be noticed that the indirect tensile strength value of open-graded asphalt mixture decreased with the increase of NMAS, which is consistent with findings from a previous study [5].

### 5.5. Air Void Content

Table 3 shows the average air void contents of the 15 mixtures in this study. The results show that the percentage of Florida washed shell does not have a significant effect on the air void content of open-graded asphalt mixtures under the same compaction effort. However, NMAS gradation slightly affects air void content in the mixture. Nonetheless, all the mixtures are within the acceptable range of 18–25%, which is the desirable range of air void content [34,35]. Since the total porosity is greater than 15%, the water inside the asphalt mixture is more likely to flow without causing drainage problems. The results also support previous research findings that a coarser gradation in an open-graded asphalt mixture can result in a higher void content under the same compaction effort [36].

### 5.6. Permeability Test

**Figure 6:**
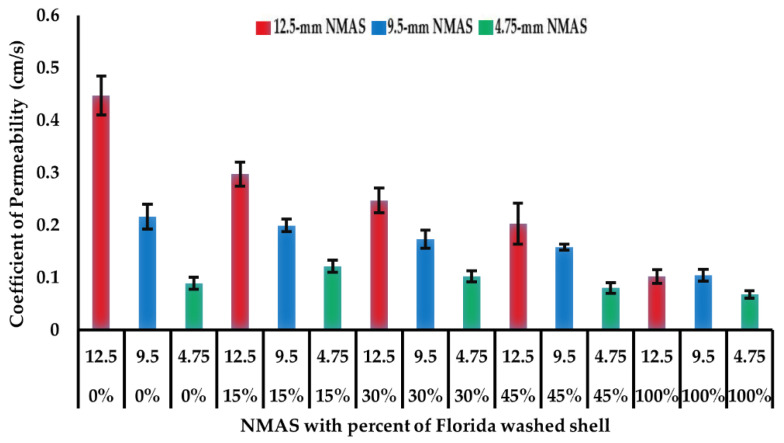
Permeability results of open-graded asphalt mixtures.

The permeability test results for the 15 open-graded asphalt mixtures are shown in Figure 6. It can be seen that the permeability increases with the increase of NMAS gradation. For the 12.5 and 9.5 mm NMAS gradations, the permeability decreases with the increase of Florida washed shell percentage. This indicates that the shape of large-sized Florida washed shell has some effect on the interconnected air void system and water conductivity in the mixture. Regarding mixtures with 4.75 mm NMAS gradations, the Florida washed shell percentage has no significant impact on the mixture permeability.

There was a noticeable disparity between the effects of shell percentage on the air void content and on the permeability. Using the 12.5 mm NMAS mixtures as an example, an increase in the percentage of Florida washed shell led to a decrease in the permeability but an increase in the air void content. This could be related to the shell impact on the three-dimensional distribution of air voids in the mixtures, as the addition of Florida washed shell may result in more isolated voids that do not contribute to effective porosity. There is still a research need to clarify the relationship between the structure and distribution of air voids in porous asphalt concrete [37].

## 6. Statistical Analysis

As a further step of analysis, a t-test was performed to determine the optimum percent of Florida washed shell as a coarse aggregate replacement for 12.5, 9.5, and 4.75 mm NMAS gradations. The optimum percent is the percent where the highest amount of Florida washed shell can be used without a significant variance in test results compared to the conventional mixture. A 5% significance level was selected in the t-test to evaluate any statistical difference between the test results from conventional and Florida washed shell mixtures. The statistical analysis and test results are shown in Table 4, Table 5 and Table 6 for the 12.5, 9.5, and 4.75 mm NMAS mixtures, respectively.

### 6.1. Statistical Analysis of Test Results of Mixtures with 12.5 mm NMAS Gradation

As can be seen from Table 4, the results of the Marshall stability test, Cantabro test, indirect tensile strength test and air void content were statistically insignificant between conventional mixtures and mixtures with 15% coarse aggregates replaced with Florida washed shell. This indicates that replacing 15% coarse aggregates in the12.5 mm NMAS open-graded asphalt mixture with Florida washed shell has no effect on strength and durability of the mixture. However, the effect on mixture permeability is significant. This is likely due to the angular shape of large-sized Florida washed shell. As a result, using 15% Florida washed shell as coarse aggregate is the optimum percent.

### 6.2. Statistical Analysis of Test Results of Mixtures with 9.5 mm NMAS Gradation

Table 5 shows the statistical analysis of the laboratory test results of mixtures with 9.5 mm NMAS gradation. As can be seen from the t-test results, the optimum percent of Florida washed shell in the mixtures as coarse aggregate is 30%, because it does not cause statistical significance in the changes of Marshall stability, Cantabro loss, indirect tensile strength, and air void content. Mixture permeability is again affected by the use of Florida washed shell from a statistical significance perspective. The average change in the mixture permeability (from 0.22 to 0.17 cm/s), however, is minor from an engineering application perspective.

### 6.3. Statistical Analysis of Test Results of Mixtures with 4.75 mm NMAS Gradation

Table 6 shows the statistical analysis of the laboratory test results of mixtures with 4.75 mm NMAS gradation. It can be seen from the t-test results, the optimum percent of Florida washed shell in the mixtures as coarse aggregate is 45%, because it does not cause statistical significance in the changes of Marshall stability, Cantabro loss, air void content, indirect tensile strength, and permeability. Although 30% of Florida washed shell mixture gave statistically insignificant results, the optimum is 45% since more Florida washed shell was utilized and more natural aggregates can be preserved.

## 7. Conclusions

This study investigated and evaluated the use of Florida washed shell in open-graded asphalt mixtures through laboratory experiments on mixture performance. Three aggregate open gradations of different nominal maximum aggregate sizes (NMASs) (i.e., 4.75, 9.5, and 12.5 mm), one Superpave performance-graded asphalt binder (PG 76-22), and granite aggregates were included in the study to prepare the asphalt mixture specimens. Coarse granite aggregates were replaced with Florida washed shell at various percentages from 0 to 100%.

Test results showed that replacing the coarse aggregates with Florida washed shell for 4.75 mm NMAS gradation up to 45% provides mixture performance similar to that of a conventional mixture. Additionally, 15% and 30% of Florida washed shell as coarse aggregate were the optimum contents for 12.5 mm and 9.5 mm NMAS gradations, respectively. Finally, 4.75 mm NMAS gradation is recommended for the open-graded asphalt mixtures for better durability, stability, and strength without much loss of permeability. It has to be noted that the above conclusions are based on the test results covered in the scope of work in this study. With additional considerations such as the functional performance (e.g., skid resistance) of the open-graded asphalt mixtures in the field, the optimum contents of shell may need to be further adjusted.

## Figures and Tables

**Table 1:** Gradation and binder content for different open-graded asphalt mixtures.

Sieve Size (mm)	NMAS 12.5 (% Passing)	NMAS 9.5 (% Passing)	NMAS 4.75 (% Passing)	Note
19	100	100	100	For granite aggregate, 15, 30, 45, and 100% of each aggregate size of coarse aggregate between sizes 2.36 and 12.5 mm are substituted with Florida washed shell.
12.5	92.5	100	100	
9.5	65	95	100	
4.75	20	32.5	90	
2.36	7.5	12.5	13	
1.18	5	5	11	
0.6	5	5	9	
0.3	4	4	7	
0.15	3	3	5.5	
0.075	3	1.5	4.5	
Asphalt Content (%)	5.5	6.0	7.5	By mass of aggregate.

**Table 2:** Physical properties of granite aggregate and Florida washed shell.

Property	Value		Method
	Granite Aggregate	Florida Washed Shell	
Loss Value of Aggregate, %	14.9	28.4	AASHTO T 96
Bulk Specific Gravity	2.677	2.600	AASHTO T 85 AASHTO T 85 AASHTO T 85 AASHTO T 85
Bulk SSD Specific Gravity	2.691	2.669	
Apparent Specific Gravity	2.716	2.792	
Water Absorption, %	0.54	2.64	

**Table 3:** Air void content of open-graded asphalt mixtures.

Type of Mixture		Specification [35]	Air Void Content %
NMAS	Percent of Florida Washed Shell		
12.5 mm	0		22.0
	15		21.7
	30		21.1
	45		20.7
	100		24.2
9.5 mm	0		20.3
	15	18–25%	20.9
	30		20.4
	45		20.0
	100		23.0
4.75 mm	0		18.3
	15		19.8
	30		19.0
	45		18.1
	100		19.3

**Table 4:** Statistical analysis of laboratory test results from open-graded asphalt mixtures of 12.5 mm NMAS.

Mixture Property	Value (Mean)	Standard Deviation	Value (Mean)	Standard Deviation	*p*-Value	t-Stat	Is there any Statistical Difference between the Test Results from Conventional and Florida Washed Shell Mixtures at a 95% Confidence Level?
	0% Florida washed shell		15% Florida washed shell				
	(conventional mixture)		as coarse aggregate				
Marshall (kN)	34.49	2.79	37.34	3.50	0.16612	−1.10220	No
Cantabro Loss (%)	44.89	6.77	41.50	8.96	0.31432	0.52296	No
ITS (kPa)	735.73	45.25	725.50	11.93	0.39245	0.29833	No
Air Void (%)	22.02	0.79	21.69	0.36	0.27586	0.64899	No
Permeability (cm/s)	0.45	0.04	0.30	0.02	0.00206	5.93026	Yes
	0% Florida washed shell		30% Florida washed shell				
	(conventional mixture)		as coarse aggregate				
Marshall (kN)	34.49	2.79	38.98	1.01	0.02936	−2.62140	Yes
Cantabro Loss (%)	44.89	6.77	29.80	3.18	0.01251	3.49433	Yes
ITS (kPa)	735.73	45.25	832.03	35.07	0.02175	−2.91400	Yes
Air Void (%)	22.02	0.79	21.12	0.74	0.11092	1.44549	No
Permeability (cm/s)	0.45	0.04	0.25	0.02	0.00072	7.83205	Yes
	0% Florida washed shell		45% Florida washed shell as coarse aggregate				
	(conventional mixture)						
Marshall (kN)	34.49	2.79	34.81	1.93	0.43870	−0.16440	No
Cantabro Loss (%)	44.89	6.77	29.59	4.30	0.01490	3.30456	Yes
ITS (kPa)	735.73	45.25	907.50	40.51	0.00402	−4.89930	Yes
Air Void (%)	22.02	0.79	20.70	0.21	0.02480	2.78431	Yes
Permeability (cm/s)	0.45	0.04	0.20	0.04	0.00073	7.79585	Yes
	0% Florida washed shell		100% Florida washed shell				
	(conventional mixture)		as coarse aggregate				
Marshall (kN)	34.49	2.79	29.03	1.14	0.01748	3.13686	Yes
Cantabro Loss (%)	44.89	6.77	22.81	1.29	0.00258	5.55000	Yes
ITS (kPa)	735.73	45.25	922.20	5.66	0.00105	−7.08330	Yes
Air Void (%)	22.02	0.79	24.48	1.01	0.01452	−3.33210	Yes
Permeability (cm/s)	0.45	0.04	0.10	0.01	5.5 × 10^−5^	15.15230	Yes

**Table 5:** Statistical analysis of laboratory test results from open-graded asphalt mixtures of 9.5 mm NMAS.

Mixture Property	Value (Mean)	Standard Deviation	Value (Mean)	Standard Deviation	*p*-Value	t-Stat	Is there Any Statistical Difference between the Test Results from Conventional and Florida Washed Shell Mixtures at a 95% Confidence Level?
	0% Florida washed shell		15% Florida washed shell as coarse aggregate				
	(conventional mixture)						
Marshall (kN)	40.30	1.94	40.17	1.91	0.46711	0.08786	No
Cantabro Loss (%)	19.92	5.15	12.18	2.42	0.03889	2.35857	Yes
ITS (kPa)	861.54	38.95	859.88	42.60	0.48133	0.04981	No
Air Void (%)	20.25	0.44	20.86	0.38	0.07286	−1.80310	No
Permeability (cm/s)	0.22	0.02	0.20	0.01	0.15981	1.13544	No
	0% Florida washed shell		30% Florida washed shell as coarse aggregate				
	(conventional mixture)						
Marshall (kN)	40.30	1.94	37.95	0.95	0.06620	1.88570	No
Cantabro Loss (%)	19.92	5.15	16.67	1.09	0.17212	1.07163	No
ITS (kPa)	861.54	38.95	848.03	18.21	0.30763	0.54414	No
Air Void (%)	20.25	0.44	20.40	0.49	0.35904	−0.38760	No
Permeability (cm/s)	0.22	0.02	0.17	0.02	0.03082	2.57506	Yes
	0% Florida washed shell		45% Florida washed shell as coarse aggregate				
	(conventional mixture)						
Marshall (kN)	40.30	1.94	32.47	0.28	0.01324	3.43233	Yes
Cantabro Loss (%)	19.92	5.15	13.21	0.69	0.08377	1.68366	No
ITS (kPa)	861.54	38.95	944.10	30.97	0.05095	−2.11520	No
Air Void (%)	20.25	0.44	23.50	0.38	0.22320	0.84359	No
Permeability (cm/s)	0.22	0.02	0.10	0.01	0.00699	4.17475	Yes
	0% Florida washed shell		100% Florida washed shell				
	(conventional mixture)		as coarse aggregate				
Marshall (kN)	40.30	1.94	32.47	0.28	0.00114	6.92014	Yes
Cantabro Loss (%)	19.92	5.15	13.21	0.69	0.04445	2.23727	Yes
ITS (kPa)	861.54	38.95	944.10	30.97	0.02264	−2.87410	Yes
Air Void (%)	20.25	0.44	23.50	0.38	0.00032	−9.67000	Yes
Permeability (cm/s)	0.22	0.02	0.10	0.01	0.00091	7.35031	Yes

**Table 6:** Statistical analysis of laboratory test results from open-graded asphalt mixtures of 4.75 mm NMAS.

Mixture Property	Value (Mean)	Standard Deviation	Value (Mean)	Standard Deviation	*p*-Value	t-Stat	Is there Any Statistical Difference between the Test Results from Conventional and Florida Washed Shell Mixtures at a 95% Confidence Level?
	0% Florida washed shell		15% Florida washed shell				
	(conventional mixture)		as coarse aggregate				
Marshall (kN)	42.47	1.67	38.78	1.67	0.02707	2.69931	Yes
Cantabro Loss (%)	6.49	1.08	4.95	0.28	0.03792	2.38171	Yes
ITS (kPa)	967.45	45.60	969.78	23.17	0.47047	−0.07890	No
Air Void (%)	18.32	0.30	19.76	0.49	0.00619	−4.32650	Yes
Permeability (cm/s)	0.09	0.01	0.12	0.01	0.01302	−3.45030	Yes
	0% Florida washed shell		30% Florida washed shell				
	(conventional mixture)		as coarse aggregate				
Marshall (kN)	42.47	1.67	39.11	4.78	0.15721	1.14946	No
Cantabro Loss (%)	6.49	1.08	9.03	2.76	0.10601	−1.48380	No
ITS (kPa)	967.45	45.60	965.40	14.43	0.47218	0.07427	No
Air Void (%)	18.32	0.30	18.95	0.36	0.03894	−2.35760	Yes
Permeability (cm/s)	0.09	0.01	0.10	0.01	0.11373	−1.42440	No
	0% Florida washed shell		45% Florida washed shell as coarse aggregate				
	(conventional mixture)						
Marshall (kN)	42.47	1.67	39.28	2.15	0.08558	1.79137	No
Cantabro Loss (%)	6.49	1.08	6.16	0.48	0.32873	0.47821	No
ITS (kPa)	967.45	45.60	881.86	32.15	0.05477	2.25387	No
Air Void (%)	18.32	0.30	18.14	0.31	0.24807	0.74778	No
Permeability (cm/s)	0.09	0.01	0.08	0.01	0.17138	1.07532	No
	0% Florida washed shell		100% Florida washed shell				
	(conventional mixture)		as coarse aggregate				
Marshall (kN)	42.47	1.67	36.75	1.67	0.00694	4.18378	Yes
Cantabro Loss (%)	6.49	1.08	4.54	1.00	0.04167	2.29582	Yes
ITS (kPa)	967.45	45.60	1123.02	32.84	0.01330	-4.07920	Yes
Air Void (%)	18.32	0.30	19.35	0.22	0.00446	-4.75650	Yes
Permeability (cm/s)	0.09	0.01	0.07	0.01	0.02594	2.74085	Yes

## Data Availability

The data presented in this study are available on request from the corresponding author.
